# Implementing a Chaotic Cryptosystem by Performing Parallel Computing on Embedded Systems with Multiprocessors

**DOI:** 10.3390/e21030268

**Published:** 2019-03-09

**Authors:** Abraham Flores-Vergara, Everardo Inzunza-González, Enrique Efren García-Guerrero, Oscar Roberto López-Bonilla, Eduardo Rodríguez-Orozco, Juan Miguel Hernández-Ontiveros, José Ricardo Cárdenas-Valdez, Esteban Tlelo-Cuautle

**Affiliations:** 1 UABC, Engineering, Architecture and Design Faculty, 22860 Ensenada, Mexico; 2 ITE, Department of Electrical and Electronic Engineering, Ensenada Institute of Technology, 22780 Ensenada, Mexico; 3 CBTIS, Industrial Technological and Services Baccalaureate Center, 82017 Mazatlan, Mexico; 4 ITT, Department of Electrical and Electronic Engineering, Tijuana Institute of Technology, 22435 Tijuana, Mexico; 5 INAOE, Department of Electronics, 72840 Puebla, Mexico

**Keywords:** cryptosystem, chaotic cryptography, embedded system, parallel computing, PRNG

## Abstract

Profiling and parallel computing techniques in a cluster of six embedded systems with multiprocessors are introduced herein to implement a chaotic cryptosystem for digital color images. The proposed encryption method is based on stream encryption using a pseudo-random number generator with high-precision arithmetic and data processing in parallel with collective communication. The profiling and parallel computing techniques allow discovery of the optimal number of processors that are necessary to improve the efficiency of the cryptosystem. That is, the processing speed improves the time for generating chaotic sequences and execution of the encryption algorithm. In addition, the high numerical precision reduces the digital degradation in a chaotic system and increases the security levels of the cryptosystem. The security analysis confirms that the proposed cryptosystem is secure and robust against different attacks that have been widely reported in the literature. Accordingly, we highlight that the proposed encryption method is potentially feasible to be implemented in practical applications, such as modern telecommunication devices employing multiprocessors, e.g., smart phones, tablets, and in any embedded system with multi-core hardware.

## 1. Introduction

Chaos theory is widely used in the encryption of information because of its particular properties [1,2,3], such as the high sensitivity to initial conditions, ergodicity, randomness, and topology complexity, among others [4,5,6,7]. For instance, numerous works related to the encryption of information using methods with chaotic and hyperchaotic models have been reported in [8,9,10,11,12,13,14,15,16,17,18,19]. On the one hand, chaotic maps have been widely used for the encryption of digital images [20,21,22,23,24,25,26], because they require generally few arithmetic operations compared to continuous-time chaotic systems. Additionally, other methods of image encryption are reported in the literature, such as those based on the substitution box (S-box) or digital watermarking using algorithms with Hall property, which use the Arnold transform to scramble the data and characterized by having low computational load [27,28].

On the other hand, pseudo-random number generators (PRNGs) [4,29,30,31,32,33,34,35,36,37,38,39,40], and truly random number generators (TRNGs) [41,42,43,44,45] are important modules in the development of cryptosystems to be robust against different types of security attacks. Some of these PRNGs and TRNGs have been implemented in personal computers and in embedded systems [29,30,31,46,47,48,49,50,51,52,53].

A large number of methods that generate sequences with PRNG/TRNG and algorithms to encrypt information for the purpose of transmitting or storing information securely has been reported in the literature [1,2,3,4,5,6,7,8,9,10,11,12,13,14,16,17,18,20,21,22,23,24,25,26,29,30,31,32,33,34,35,36,37,38,39,40,41,42,43,44,45,46,47,48,49,50,51,52,53]. Most of these works use double precision in their arithmetic floating-point operations according to the IEEE 754 standard [54]. However, one of the important limitations when implementing chaotic systems in digital devices is related to their numerical precision [55]. In this manner, due to the numerical rounding and in relation to the complexity of the chaotic map dynamics, after a certain number of iterations the generator may present a certain degree of periodicity or degradation of digital chaos [56,57,58]. This effect limits digital chaotic encryption for large amounts of information, such as high-resolution images (greater than 1 megapixel), image sequence, audio, and video. Therefore, many of the proposed digital image encryption schemes are designed using chaotic maps with permutation-diffusion architecture. Although most of these schemes report good statistical properties, their speed of execution is slow due to the inherent dependence of the data of the proposed schemes. Some of these schemes are designed using complex chaotic systems that require significant computational resources to obtain the key streams for the information encryption [59].

Moreover, technology is constantly evolving with significant advances in the new generation of embedded systems with greater computing power including multi-core processors, for that reason, new threats and vulnerabilities that compromise security information in telecommunications systems are being devised [60]. Recently, several attacks and cryptanalysis to cryptosystems have been reported in the literature [61,62,63,64,65,66], therefore, it is important to continue with the development of new cryptosystems with greater complexity and efficiency, that is, to increase their security, such as key space, entropy, resistance against differential and statistical attacks, among others. Thus, the great technological advances and new emerging technologies have allowed an exponential increase in practical applications for the Internet of Things (IoT), for which it is expected that by the year 2020, more than 50,000 million devices, machines, and systems with digital communication technology through the use of embedded systems will be connected to the Internet [67,68,69]. By interconnecting machines and systems with the Internet, IoT devices can be connected wirelessly or directly via Ethernet cable through a network switch or access point [70]. However, these services and multiple electronic applications that carry out the exchange of confidential information through public telecommunications channels, such as the Internet, and that use electronic devices or embedded systems with digital technology [71], put privacy and confidentiality at risk of the information that is processed. Therefore, the cryptographic security in these systems is an integral and indivisible part of this evolutionary process, thus the necessity to constantly develop new methods, systems, and techniques to protect confidential information and to guarantee a greater cryptographic security in modern telecommunications systems [13,72,73].

Meanwhile, parallel computing is a form of computation in which many instructions are executed simultaneously [74]. It operates on the principle that large problems can often be divided into smaller ones, which are then solved simultaneously (parallel). There are different types of parallel computing: bit-level parallelism, instruction level parallelism, data parallelism, and task parallelism. Parallelism has been employed for many years, especially in high-performance computing; however, interest in this field has grown lately due to the physical hardware that limits the increase of frequency of CPUs [41,59,74,75]. In addition, the consumption of energy and consequently the generation of heat from computers constitutes a focal point in the technological development of recent years [75,76]. Therefore, parallel computing has become a dominant paradigm in computer architecture, mainly in the form of multi-core processors [76].

From our knowledge and based on the reviewed literature, few works report the use of parallel computing for the encryption of information, see for example [15,41,44,59,77,78,79,80,81,82], but nevertheless, there are not any reported works in which profiling techniques and parallel computation are used for the encryption of high-resolution digital images using PRNGs on a cluster of embedded systems with multiprocessors. Except the work [41] reports a parallelizable chaos-based TRNG implemented on mobile device, but is not reported any implementation of parallel encryption on embedded system. Therefore, this paper proposes to use a different hardware from that commonly reported in the literature. The experiments are performed in a cluster with six System on Chip (SoC) Raspberry Pi 3, which allows us to physically integrate up to 24 cores, to run as many feasible processes that use parallel computing, and thus improve the efficiency of the new generation of embedded cryptosystems. Additionally, the method reported by [73] is used for the encryption of information using multiple-precision arithmetic [83]. The Python programming language [84] is used to process several significant decimals higher than that reported in the works [1,2,3,4,5,6,7,8,9,10,11,12,13,14,16,17,18,20,21,22,23,24,25,26,29,30,31,32,33,34,35,36,37,38,39,40,41,42,43,44,45,46,47,48,49,50,51] and which are based on the IEEE 754 standard [54], typically used in computers and FPGAs. It is important to emphasize that Python is a scientific programming language [84] with the following advantages: open-source, free license, and multiplatform. Under this perspective, the main contribution of this paper is that through the cluster integrated by six embedded systems, the feasible processes of parallelization are executed to improve the efficiency of the proposed cryptosystem by executing high-precision numerical operations under the scheme described by [73]. Additionally, the proposed cryptosystem must comply with the basic requirements of any chaos-based cryptographic system [85], such as the NIST SP 800-22 statistical tests designed for cryptographic modules [86,87] and other well-known attacks in the literature, such as Number of Pixels Change Rate (NPCR) and Unified Average Changing Intensity (UACI) differential attacks [88], entropy [89,90,91], key space [92,93], which are commonly applied to the cryptosystems.

The rest of this paper is organized as follows: Section 2 describes the proposed PRNG and dynamic keys generator. Section 3 describes the implementation of the proposed high-precision cryptosystem using parallel computing and profiling. Section 4 provides a security analysis according to [85,86,87,88] and performance analysis according to Amdahl’s law [94]. The last section concludes this paper with a summary of the results achieved.

## 2. Proposed PRNG and Dynamic Keys Generator

**Figure 1:**
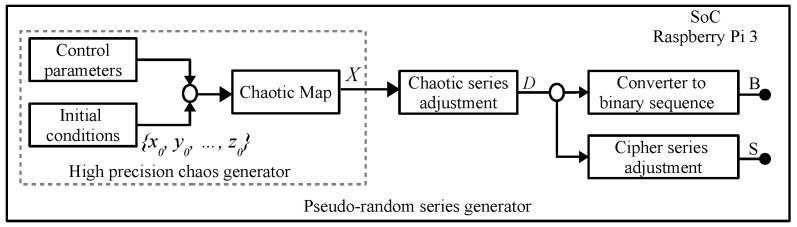
Pseudo-random number generator (PRNG) implemented in a SoC Raspberry Pi 3, taken from [73].

The PRNG and dynamic keys generator that is implemented in this paper is shown in Figure 1, both corresponds to the method reported by [73], which use multiple precision. In this work we introduce a method to encrypt/decrypt color images applying profiling techniques and parallel computing, with the aim to improve the cryptosystem efficiency to be implemented on embedded systems with multi-cores that have limited computational resources. As an example the Tinkerbell map [73,95,96] is defined by (1), which remains in a chaotic regime for the ranges of its control parameters 0.84<a≤0.9, −0.61<b<−0.59, 1.9<c≤2 and 0.45<d≤0.5, where $-1.25\leq x_{n}\leq \;0.55$ and $-1.6<y_{n}\leq 0.55$, and initial conditions $x_{0}$ and $y_{0}$ that correspond to points that are within the space of the chaotic attractor, such as $x_{0}=-0.72$ e $y_{0}=-0.64$ [96].

(1) $$\begin{array}{ccc} x_{n+1} & = & x_{n}^{2}-y_{n}^{2}+ax_{n}+by_{n},\\ y_{n+1} & = & 2x_{n}y_{n}+cx_{n}+dy_{n}. \end{array}$$

The generated pseudo-random sequences consider two attributes: (i) the number of significant decimals is predefined to establish their level of precision np, for example np=99 and (ii) each sequence is generated with different initial conditions and recurrent points of its attractor. For example, taking as initial conditions $x_{0}=-0.72$, $y_{0}=-0.64$ and iterating k=2500 times (one can choose a number greater than 2500), if (1) considers a numerical precision np=99, one gets [73]:

$x_{1}$ = −0.5044175778616422926572575875229294547046448096553074629030668113991951…
$y_{1}$ = −0.3460286548462436652145266889485621403916728515023067284157645131661259…

From these values, a new level of precision and $np_{x_{0}}$ truncation of significant decimals for the new initial $x_{0}^{\prime }$ and $y_{0}^{\prime }$ are predefined. For example, if $np_{x_{0}}$ = 50 significant decimals, then is obtained:

$x_{0}^{\prime }$ = −0.50441757786164229265725758752292945470464480965530
$y_{0}^{\prime }$ = −0.34602865484624366521452668894856214039167285150230.

**Figure 2:**
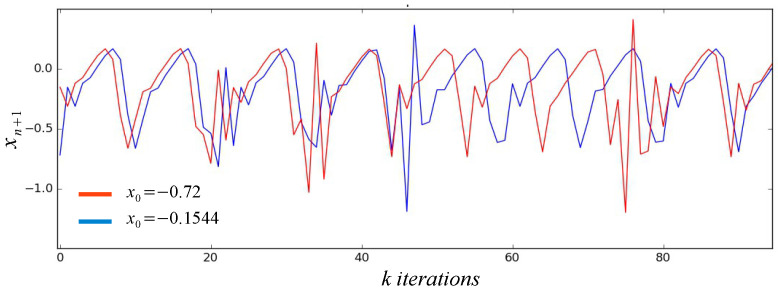
High-precision chaotic trajectories with pseudo-random initial conditions.

This procedure guarantees that the new values of initial conditions are within the space of the chaotic attractor and are valid in the long term for new initial conditions, that is, new dynamic keys are generated for the encryption of large amounts of information. When generating numerical values for the initial conditions very different from each other, they produce chaotic series with different dynamic behaviors without losing their properties, which makes them suitable for use in the encryption of images regardless of their level of resolution. Figure 2 shows an example of the chaotic trajectories of $x_{n+1}$. It is observed that due to the sensitivity to the initial conditions presented by the chaotic systems, different trajectories are generated from the first iteration.

### Proposed Pseudo-Random Bit Generator (PRBG)

**Figure 3:**
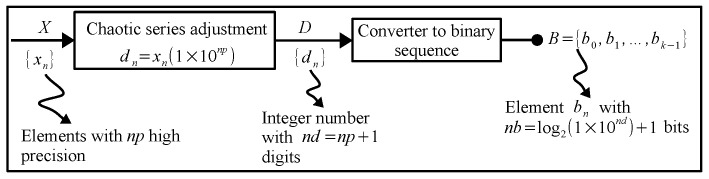
Block diagram of the pseudo-random bit series generator using HPCG, taken from [73].

The proposed PRBG shown in Figure 3, is based on the high-precision chaos generator (HPCG) described in [73]. In this paper, we show the use of parallel computing techniques for the simultaneous generation of bit sequences, which allows the cryptosystem to be more efficient when using its multiple cores. In relation to Figure 3, each pseudo-random sequence of bits is obtained by converting each number of the chaotic series generated into a binary equivalent through the [Chaotic series adjustment] and [Converter to binary sequence] blocks. The binary equivalent that results in each iteration is concatenated each time and stored in a resulting bit string identified with letter *B*. In this way, a pseudo-random *B* string of bits is generated for each equation contained in the chaotic map.

## 3. Proposed Parallel Encryption Method

**Figure 4:**
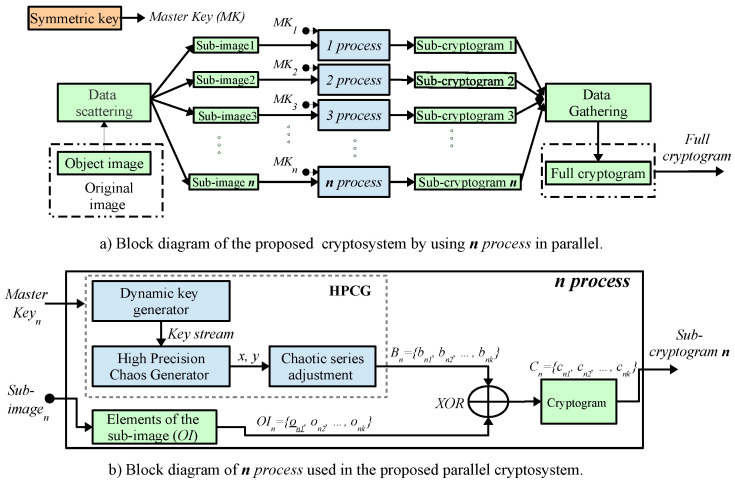
Block diagram of image encryption method using parallel computing.

The proposed system for encryption and decryption of digital color images is shown in Figure 4. It is based on the method of stream encryption with *Symmetric key* [97], and in the method for generation of pseudo-random numbers using multiple-precision arithmetic reported in [73]. In relation to Figure 4a), the *Original image OI (Object image)* goes through a data distribution stage that is illustrated in the [Data scattering] block and that corresponds to divide the image into *n* blocks (*sub-images*), $OI_{1}=\left\{o_{11},o_{12},\ldots ,o_{1k}\right\}$, $OI_{2}=\left\{o_{21},o_{22},\ldots ,o_{2k}\right\}$, …, $OI_{n}=\left\{o_{n1},o_{n2},\ldots ,o_{nk}\right\}$, where $o_{nk}$ corresponds to *k*-pixel of the plain image ($OI_{n}$). Each of these *sub-images* are sent to each *n process* of the embedded system cluster, where they are encrypted simultaneously (parallel), as shown in Figure 4a). In Figure 4b) each *n process* encrypts the $o_{nk}$ pixels with the corresponding byte to the associated encryption series $B_{1}=\left\{b_{11},b_{12},\ldots ,b_{1k}\right\}$, $B_{2}=\left\{b_{21},b_{22},\ldots ,b_{2k}\right\}$, …, $B_{n}=\left\{b_{n1},b_{n2},\ldots ,b_{nk}\right\}$, through the simple logical operation XOR. Subsequently, each of the *sub-cryptograms* achieved $C_{1}=\left\{c_{11},c_{12},\ldots ,c_{1k}\right\}$, $C_{2}=\left\{c_{21},c_{22},\ldots ,c_{2k}\right\}$, …, $C_{n}=\left\{c_{n1},c_{n2},\ldots ,c_{nk}\right\}$, are sent to the [Data Gathering] stage, where all the encrypted information is integrated to obtain the *Full cryptogram C*. To execute the scheme of the Figure 4a) for data processing in parallel with collective communication, it is proposed to use the Message Passing Interface (MPI) library reported in [98,99] commonly used in computers, nevertheless, in this paper, it is implemented in a cluster of six embedded systems with multi-core processors, which is described in Section 3.5. When using the logical operation XOR as an encryption operator and due to its duality property, the same method can be used to encrypt and decrypt digital images [73].

### 3.1. Profiling Algorithms Using Python

A profiler is a program that analyzes and collects information about the behavior of another object program during its execution. The type of information that is analyzed includes the processing times of the program’s subroutines and the number of times each subroutine is called. The above, with the purpose of allowing the developer to optimize the program code to improve the speed in the execution of the complete program, adjusting the code design implementing adjustment techniques and, if possible, the implementation of parallel processing techniques. The profiling tool used in this work is the *“line-by-line”* profiling tool known as **Line_Profiler** for Python. **Line_Profiler** is supported by the tag **@profile** to identify the function of the program to be profiled and perform the corresponding analysis. The performance of the “Encrypter” with the profiling tool is done by means of the command: ***kernprof -l -v Encrypter.py***, run from the GNU Linux console. The **-v** parameter of the ***kernprof*** command allows the user to observe the profiling analysis report as an immediate response to the execution of the previous command in the command terminal. Otherwise, the profiling tool stores the corresponding analysis information in a binary file with extension **.lprof** with the same name as the Python code file. The complete report can be observed executing the report file through: ***python -m line_ profiler Encrypter.py.lprof***. The following report is an example that corresponds to the profiling analysis of the encrypt() function, considering the “landscape.png” file as the object image. The image to be encrypted is a high-resolution color image in 32-bit format with 2560×1600 pixels, that is, each pixel of the image is composed of 4 elements of 8 bit giving a total of 16,384,000 bytes to be encrypted.


Wrote profile results to Encrypter.py.lprof
Timer unit: 3.79968e-07 s
 
Total time: 862.992 s
File: Encrypter.py
Function: encrypt at line 5
Line #      Hits         Time  Per Hit   % Time  Line Contents
==============================================================
     4                                           @profile
     5                                           def encrypt():
     6         1       146078 146078.0      0.0          foto = Image.open(’land…
     7         1       761832 761832.0      0.0          foto = np.array(foto)
     8         1           63     63.0      0.0          ren = foto.shape[0]
     9         1            7      7.0      0.0          col = foto.shape[1]
    10         1            6      6.0      0.0          cap = foto.shape[2]
    11         1            8      8.0      0.0          vector=ren*col*cap
    12         1           97     97.0      0.0          foto=np.reshape(foto,(v…
    13         1           98     98.0      0.0          a= Decimal(’0.9’)
    14         1           11     11.0      0.0          b= Decimal(’-0.6013’)
    15         1            9      9.0      0.0          c= Decimal(’2.0’)
    16         1            9      9.0      0.0          d= Decimal(’0.5’)
    17         1            8      8.0      0.0          x0= Decimal(’-0.72’)
    18         1            8      8.0      0.0          y0= Decimal(’-0.64’)
    19         1            5      5.0      0.0          ret=0
    20         1            4      4.0      0.0          npx0=8
    21         1            4      4.0      0.0          np_prec=99
    22         1           15     15.0      0.0          getcontext().prec=npx0
    23         2           12      6.0      0.0          while (ret<1):
    24         1           69     69.0      0.0              x1 = Decimal((x0*x0)…
    25         1           14     14.0      0.0              y1 = Decimal((2*x0*y…
    26         1            5      5.0      0.0              x0=x1
    27         1            6      6.0      0.0              y0=y1
    28         1            6      6.0      0.0              ret=ret+1
    29         1            6      6.0      0.0          getcontext().prec=np_prec
    30         1            6      6.0      0.0          j=0
    31  16384001     78137141      4.8      3.4          while(j<vector):
    32  16384000    439917303     26.9     19.4                  x1 = Decimal((x0*…
    33  16384000    351247125     21.4     15.5                  y1 = Decimal((2*x…
    34  16384000     87992069      5.4      3.9                  x0=x1
    35  16384000     79512478      4.9      3.5                  y0=y1
    36  16384000    300653689     18.4     13.2                  x1=x1*Decimal(10**…
    37  16384000    136258513      8.3      6.0                  x1=Decimal.to_inte…
    38  16384000     91137986      5.6      4.0                  if x1<0:
    39  10906047     83757897      7.7      3.7                          x1=x1*(-1)
    40  16384000    185584508     11.3      8.2                  s1=int(x1%255)
    41  16384000    337097363     20.6     14.8                  foto[j]= foto[j]^s1
    42  16384000     93981721      5.7      4.1                  j=j+1
    43         1           77     77.0      0.0          foto=np.reshape(foto,(ren,c…
    44         1          456    456.0      0.0          foto=Image.fromarray(foto)
    45         1      5036187 5036187.0      0.2          foto.save(’output.png’)
        

The report initially displays the value of $3.79968e^{-07}$ s as timer unit, and it is observed that the total execution time of the encrypt() function is 862.992 s. Additionally, the report indicates the execution time of each instruction per program code line. From the results reported in [73], it is observed that the series for the state $y_{n+1}$ also present high levels of randomness, so that they are potentially viable to be used as a cipher series. In this paper, the proposed method also includes the chaotic series $y_{n+1}$, and it is estimated that the encrypt() function reduces the total time, as T(1)=639 s, corresponding to the iterations of the chaotic map when n=1 process. Therefore, with the information obtained through the profiling tool, one can identify the instructions that are executed a greater number of times, thus requiring a greater amount of time. This allows identification of in a specific way, where to implement code optimization adjustment techniques, or to divide the main program in one part with sequential processing and another parallelizable.

### 3.2. Parameters of Parallel Processing

Profiling analysis can determine that the function can be divided into a sequential part and a parallelizable part. There are a series of parameters to evaluate the parallel processing as the performance improvement factor or “speed-up” and the efficiency of the performance. If O(n) is defined as the number of elementary operations performed by a system with *n* process, and T(n) as the execution time in unit steps of time, then T(n)<O(n), if *n* process perform more than one operation per unit of time. The performance improvement factor S(n) for *n* process is defined by (2), and the efficiency of the E(n) system, for a system with *n* processes, is determined by (3).

(2) $$\begin{array}{c} S\left(n\right)=\frac{T(1)}{T(n)}, \end{array}$$

(3) $$\begin{array}{c} E\left(n\right)=\frac{S(n)}{n}=\frac{T(1)}{n\cdot T(n)}. \end{array}$$

The lowest system’s efficiency E(n)→0 occurs when all instructions in the object program are executed sequentially in a single processor system. The maximum system efficiency E(n)=1 corresponds to the case in which all the processors of the system are being completely used during the execution of the program. Another parameter for the evaluation of systems with parallel processing is scalability. It is said that a system is scalable for a certain number of *n* process, if the efficiency of the system E(n) is constant and at all times greater than a factor of 0.5 [100]. In practice, scalable systems can be divided into several *n* process from which the efficiency of the system begins to decrease. In the analyzed program from the profiling report, of the total instructions, the 99.7% is potentially parallelizable (lines 13 to 42 of profiling report) and 0.3% is executed sequentially. Therefore, according to the analysis of the profiling report, if T(1)=639 s the values of S(n) and E(n) for n=2 are:

$$\begin{array}{ccc} T(1) & = & 639s=99.7\%+0.3\%=637.08s+1.92s,\\ S(2) & = & T(1)/T(2)=639s/(318.54s+1.92s)=639s/320.46s=1.994,\\ E(2) & = & S(2)/2=T(1)/2*T(2)=1.9940/2=0.9970. \end{array}$$

**Figure 5:**
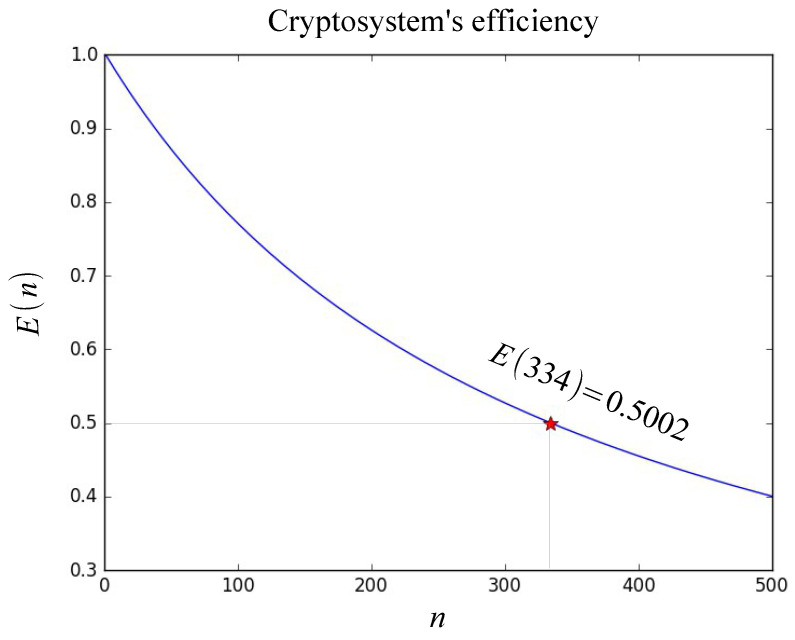
Characteristic curve of cryptosystem’s efficiency E(n) with α=0.3%.

A first approximation to the *n* process among which the analyzed program can be established from Equation (3), that is,

$$\begin{array}{ccc} E(n) & = & 50\%,\;i.e.\;0.5, \end{array}$$

if T(1)=639 s, and is considered to be 100%,

$$\begin{array}{ccc} T(1) & = & 100=\alpha +\beta_{p}\left(n\right)=0.3+99.7,\\ T(n) & = & \alpha +\beta_{p}\left(n\right)=\alpha +\left(\beta_{p}\left(1\right)/n\right)=\alpha +\left(100-\alpha \right)/n, \end{array}$$

then,

$$\begin{array}{ccc} 0.5 & = & 100/(n(\alpha +(100-\alpha )/n)),\\ 0.5 & = & 100/((n-1)\alpha +100),\\ n-1 & = & ((100/0.5)-100)/\alpha ,\\ n & = & 100/\alpha +1=334.33, \end{array}$$

where, α is the sequential time, and $\beta_{p}$ is the parallel time. Figure 5 shows the graph of the efficiency values of the system E(n) of the “Encrypter.py” program. It is observed that for n>334 the efficiency of the system is less than 0.5.

In relation to [100], the performance factor can be classified into three different types: (i) based on whether the load or problem size is fixed and what is intended to be reduced is the problem execution time, (ii) on the basis that there is a certain time to execute the problem or that it is intended to increase the size of the machine and, (iii) that applies to scalable problems where the main limitation in the execution process is the system memory. In this paper, a problem in which the computational load or problem size is fixed, and is intended to reduce the execution time of encryption process. Therefore, it is necessary to perform an analysis of the performance factor based on Amdahl’s law [94]. Amdahl’s law considers problems where the objective is to distribute the fixed load in more process to decrease the total execution time. In this case, the Amdahl performance improvement factor ($S_{n}$) described by (4), is determined by the percentage of the sequential time of the algorithm which is identified as α. The percentage of parallel time is identified with $\beta_{p}=1-\alpha$,

(4) $$\begin{array}{c} S_{n}=\frac{n}{1+(n-1)\alpha }. \end{array}$$

**Figure 6:**
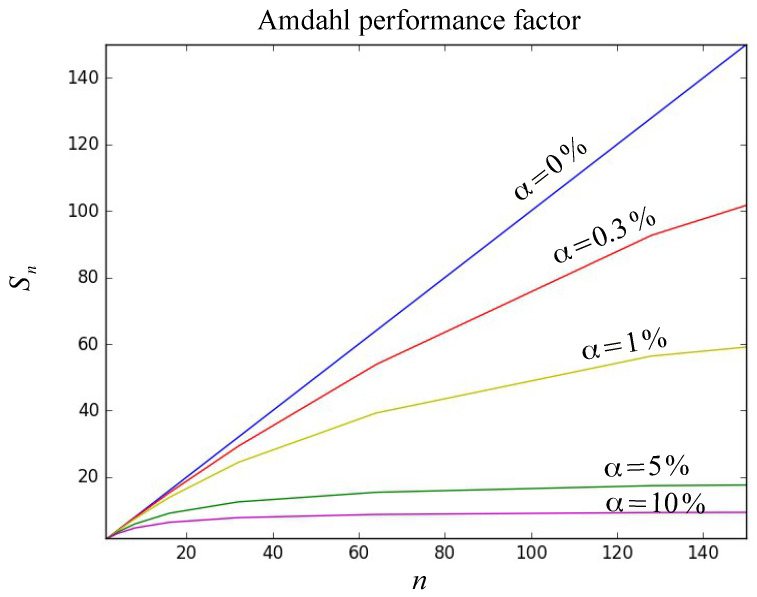
Curves of performance factor $S_{n}$ for different α values compared to the $S_{n}$ curve for the proposed cryptosystem with α=0.3%.

On the proposed cryptosystem, the percentage of sequential time α=0.3% implies that $S_{n}$ approaches asymptotically at 1/α as the number of *n* process increases. The graph of Figure 6 shows the characteristic curve of Amdahl performance factor considering a sequential part α of 0.3% compared to the characteristic curve when the parallelizable part is zero or α=0%. Additionally, and for comparison purposes, the curves are shown for different values of α where it is observed that as the percentage of sequential time increases, the “speed-up” value of Amdahl performance factor begins to decline to a great extent.

In the analysis performed it is considered that the process is under ideal conditions of computational resource of software and hardware, that is to say, at the time of executing the algorithm each time, considering different numbers of process, each process works under the same conditions and with the same computational resources. In the realization, another factor that is considered in the study of parallel processing is the communication between processors. In the cryptosystem, the computational load or size of the problem corresponds to the image that will be encrypted or decrypted. Considering that when carrying out the parallelization process the computational load is evenly distributed among the total number of processors executing the algorithm, then, there is a distribution time for the image in equal parts to be encrypted and a collection time of the sub-cryptograms to obtain the full cryptogram. Due to this, the total time in the execution of the algorithm is affected by the distribution and collection of the data load. Presently, there are tools that perform collective communication optimizing the distribution and load collection times among the processors, such as the MPI tool for Python, which provides collective communication functions that optimize the distribution and data collection times and information between process [98,99].

### 3.3. Implementing the MPI Library Using Collective Communication in the Algorithm

The code fragment shown in Algorithm 1, describes the setup of the system’s processors involved in the cryptosystem. In addition to a part of the sequential processing of the algorithm α, this part of the sequential processing is responsible for reading from the file of original image to encrypt or decrypt, identified with the name of “image.png” and the re-arrangement of the data in an equitable manner for distribution through collective communication.

**Algorithm 1** First sequential time (α) 1: from mpi4py import MPI 2: import numpy as np 3: from PIL import Image 4: from decimal import * 5: comm=MPI.COMM_WORLD 6: size=comm.Get_size()        # Number of processors 7: rank=comm.Get_rank()       # Processor ID  8: if rank == 0: 9: imagen=Image.open(’image.png’)    # Read original image 10: imagen=np.array(imagen)      # Convert the object image into an array 11: ren=imagen.shape[0]       # Size of the array (rows) 12: col=imagen.shape[1]       # Size of the array (cols) 13: cap=imagen.shape[2] 14: n_ren=np.int(ren/size)            # Resize the array 15: foto=np.reshape(imagen,(size,n_ren,col,cap))   # for scattering data

The instruction “size=comm.Get_size()” identifies the total number of processors included in the cryptosystem, and the instruction “rank=comm.Get_rank()”, identifies each processor. The reading of original image is done through the “Image.open()” function provided by the image processing tool for Python PIL. The “Image.open()” function returns an object of the “Image” class, therefore, the object is converted to an “array” type to obtain a matrix for processing with the “numpy” tool. The aforementioned instructions are executed by a first processor which is identified as rank=0 and performs the functions of the main processor (coordinator). In other words, the main processor is responsible for reading the data to be processed, for the equitable data distribution between the different processors, and for collecting the information to provide the final result, i.e., full cryptogram. Once the plain image is read and distributed, each processor (including the main processor) executes the encryption method described in Figure 4b). In the [Symmetric key] block, the values corresponding to the encryption master key are set. In the [Dynamic key generator] block, the initial conditions for each processor are generated independently this, in order that each processor count with a different encryption key. In section [Data scattering], the scatter() function is used to optimize the time in data distribution. Additionally, once the equitable distribution is made, each processor has the corresponding sub-image to be encrypted, therefore, for the purpose of optimizing the cryptographic method, the data is re-arranged in a one-dimensional array. Subsequently, the instructions for the blocks [HPCG], [Chaotic series adjustment] and [XOR] are executed. Once the cryptographic process has been performed, the instructions in the [Data gathering] section are executed to send the result to the main processor, the sending of information is done through the data collection function “comm.gather()”. It is important to highlight that the functions “comm.gather()” and “comm.scatter()” work in a corresponding way, that is, the format of the sending data must be the same as the receiving format, therefore, it is necessary to perform a re-arrangement of the resulting vector to its original matrix format. Finally, the main processor (rank=0), collects the information and converts the resulting matrix array into an object of the “Image” class to store the final result in a file identified as “output.png”. The above is done through the instructions of the code fragment shown in Algorithm 2.

**Algorithm 2** Second sequential time (α) 1: if rank == 0: 2: foto=np.array(newData) 3: foto=np.reshape(foto,(ren,col,cap)) 4: foto=Image.fromarray(foto) 5: foto.save(’output.png’) 6: fin=datetime.now() 7: print(“Done!”) 8: print(end-brgin) 9: if __name__ == ’__main__’: 10: encrypt()

### 3.4. Implementing a Machine Based on Raspberry Pi 3

**Figure 7:**
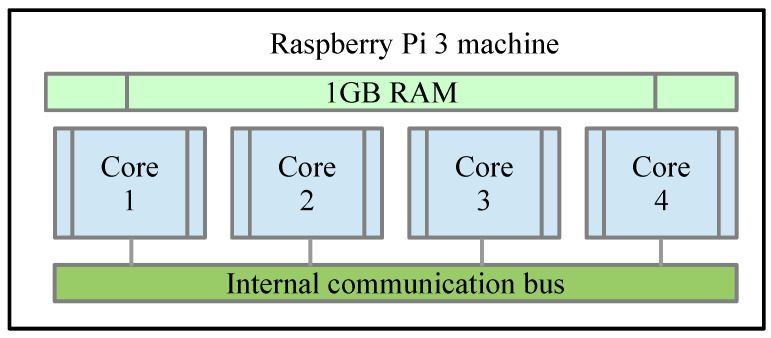
Block Diagram of Raspberry Pi 3 Machine.

The SoC Raspberry Pi 3 has a Cortex-A-53 64-bit Quad Core 1.2 GHz processor with 1 GB DDR2 RAM memory. One of the aspects considered in the study of parallel processing is the processor machine in which the algorithms are executed. The execution of the main algorithm that uses parallel processing through the use of the MPI library [98,99] and the profiling tool is carried out by means of the command: ***mpiexec -n*<n>*kernprof -l -v Encrypter.py***, where the parameter <n> indicates the number of process that the machine that executes the algorithm contains. Figure 7 shows the composition of the Raspberry Pi 3 machine where it is observed that internally has a Quad Core structure with internal communication bus and 1GB of RAM.

**Figure 8:**
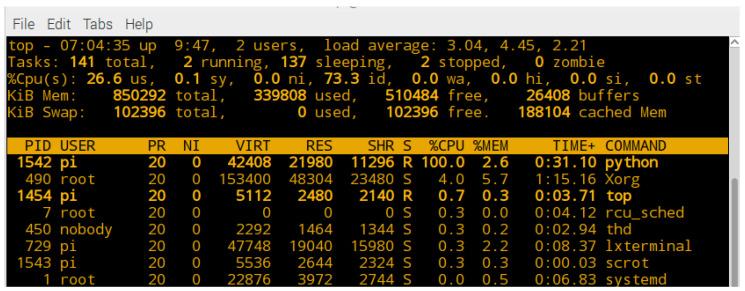
Computational resource assigned to Raspberry Pi 3 virtual machine with n=1 processor.

When executing the previous command, the Raspberry Pi 3 system creates a machine with *n* process for program execution. Figure 8 shows some of the applications and services that are running on the Raspberry Pi 3 system when executing the command for processing with 1 processor (n=1). It is observed that a virtual machine is created with a resource of %CPU=100.0 and a memory resource of %MEM=2.6 in a process identified as PID=1542.

**Figure 9:**
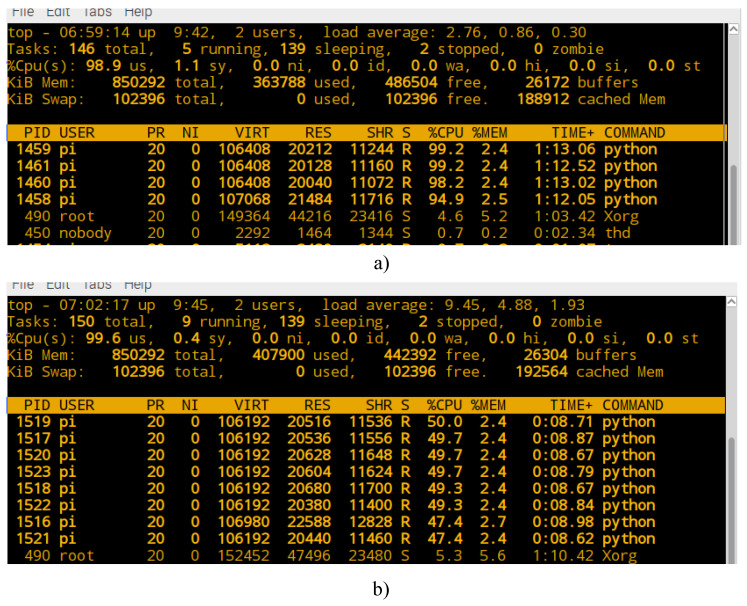
Computational resource assigned to Raspberry Pi 3 machine: (**a**) with n=4 process and (**b**) with n=8 process.

Figure 9a, shows the execution of the machine considering n=4 process, and Figure 9b shows the computational resource assigned to each processor when the proposed method is executed with n=8 process. It is observed that each processor is assigned to a computational resource of CPU different from that assigned to each processor when it increases the process’ number of the machine. Due to the above, the Amdahl performance factor ($S_{n}$) calculated and shown in the graph of Figure 6, is greatly affected because the machines do not have the same computational hardware resource for execution as the number of process increases.

From the comparison shown in Figure 9a,b, the most optimal computational resource for the Raspberry Pi 3 machine, is presented when the number of process that execute the program is n=4. Therefore, the maximum gain in runtime is obtained with n=4 processors. From n>4 process the gain in runtime decreases each time, this is because physically there are no more processors in a Raspberry Pi 3.

### 3.5. Implementing a Machine Based on a Cluster of Six Embedded Systems (FIAD Cluster)

**Figure 10:**
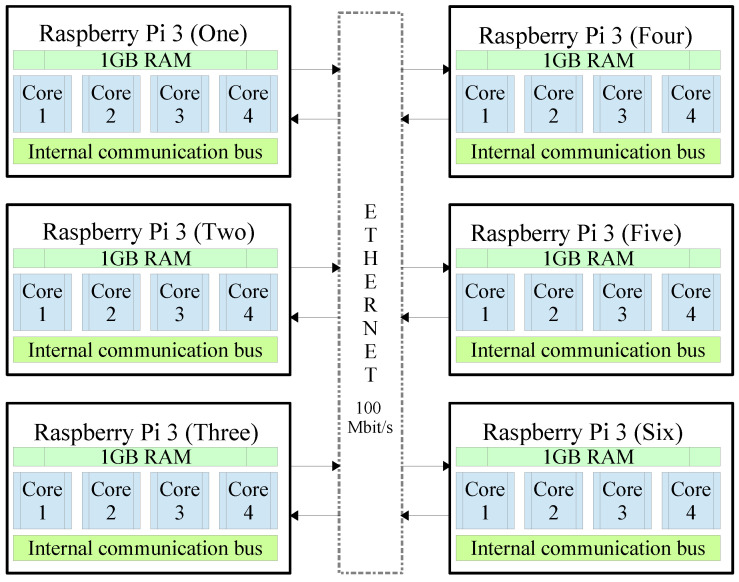
FIAD cluster machine composed of six Raspberry Pi 3.

**Figure 11:**
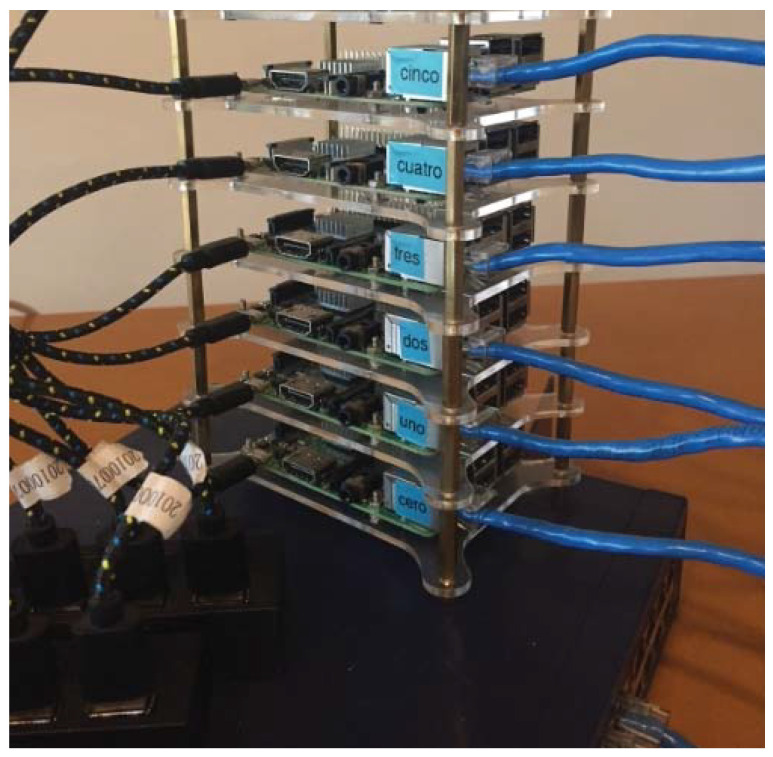
Image of the experimental arrangement of the FIAD cluster.

The FIAD cluster is composed of six Raspberry Pi 3 embedded systems and whose organization is generally described in Figure 10. It is observed that when the complete machine is integrated, a total of 24 Cores is obtained. In theory it is expected that the maximum gain will be obtained in executions of the program with n=24 process. However, in addition to the fact that each Raspberry Pi 3 system has an internal communication bus, each Raspberry Pi 3 communicates externally with the remaining 5 devices through an Ethernet communication channel at a speed of 100 Mbit/s. That way, the communication time between processes is affected. The Figure 11 shows an image of the experimental arrangement implemented for the execution of encryption tests using parallel computing on six embedded systems.

## 4. Experimental Results

To test the robustness and security of the proposed cryptosystem, the following security analyses were performed: Statistical tests of NIST for cryptographic modules, histogram analysis, entropy, differential attack tests (NPCR and UACI) and key space. The quality evaluation of the randomness is carried out to demonstrate the satisfactory security of the new proposed chaos-based cryptosystem. In addition, to test the efficiency of the cryptosystem, a performance analysis of parallel computing in information encryption is accomplished. Besides, with the purpose of testing the proposed method in a different hardware and with multiprocessing capability, it also was implemented in a computer with CPU AMD A6 4400M APU 2.7 GHz (Accelerated Processing Unit), which is a mobile dual-core processor based on the Trinity architecture, works with Windows 10 operating system and Python version 3.5.2.

### 4.1. Security Analysis

#### 4.1.1. The NIST Statistical Test

For NIST tests, each *p*-value is the probability that a perfect random number generator would have produced a sequence less random than the sequence that was tested, given the kind of non-randomness assessed by the test. If a *p*-value for a test is determined to be equal to 1, then the sequence appears to have perfect randomness. A *p*-value of zero indicates that the sequence appears to be completely non-random. A significance level (α) can be chosen for the tests. If *p*-value ⩾α, i.e., the sequence appears to be random. If *p*-value <α, i.e., the sequence appears to be non-random. For all 16 tests in the NIST suite [86] performed in this paper, the significance level (α) was set to 0.01. If a computed *p*-value is greater than 0.01, the binary sequence is accepted as random with a confidence of 99%; otherwise, it is considered to be non-random [4,86,101]. In addition, the following setup parameters are considered: Block Frequency test - block length (*M*) = 128, Non-Overlapping Template test - block length (*m*) = 9, Overlapping Template test - block length (*m*) = 9, Approximate Entropy test - block length (*m*) = 10, Serial test - block length (*M*) = 16, Linear Complexity test - block length (*M*) = 500. All these tests were performed using 1000 series (sequences) of stream length = 1,000,000 bit. Table 1 lists the comparative results of the success percentages obtained with Tinkerbell map [73,95,96] using high precision of np = 99 significant decimals. If the proportion of success is greater than 0.98, it can be concluded that the sequences pass the NIST tests, i.e., those are random sequences. These results demonstrate that the proposed PRNG can be used in cryptosystems [73].

#### 4.1.2. Histogram Analysis

**Figure 12:**
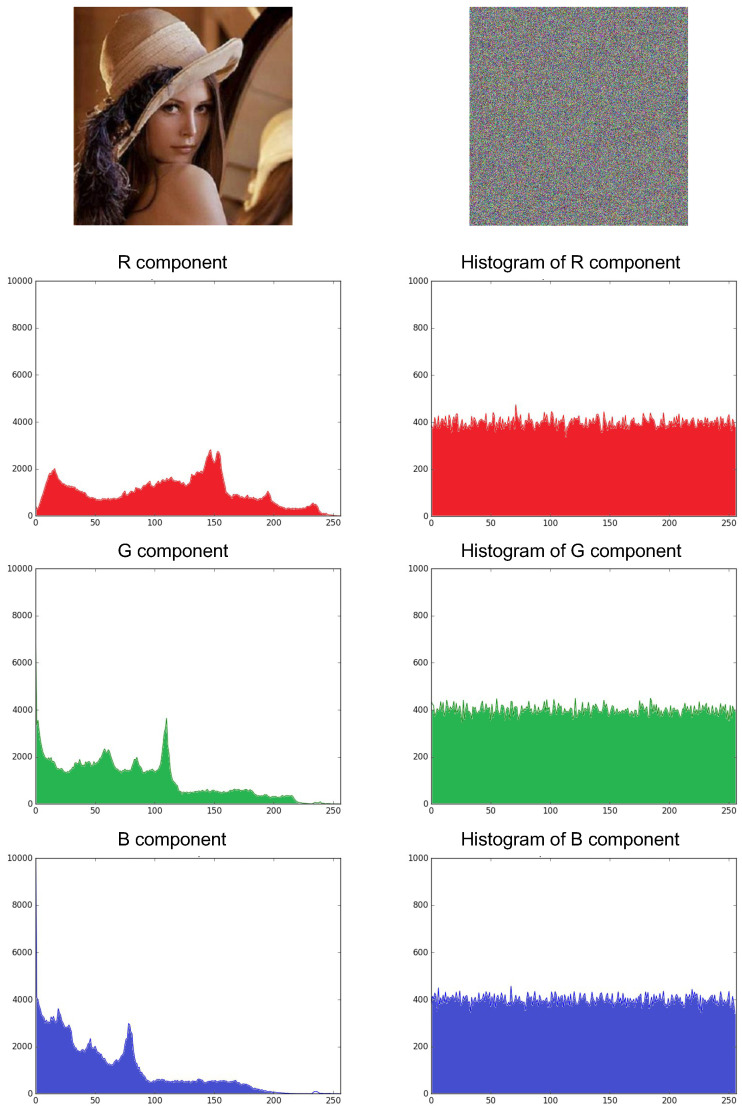
RGB Image of “Lena.png” with size 512×512×3 and cryptogram obtained using the proposed cryptosystem with the Tinkerbell map [73] with both chaotic states in the encryption method.

The histogram of a digital image is a graph that shows the number of pixels of each different intensity value found in the image. For an image with an 8-bit format, there are 256 different intensity levels [93]. The distribution of the histogram of the encrypted image is the most important. More specifically, it must hide the redundancy of the original image and no confidential information should leak from the original image or that there will be a relationship between the encrypted image and the original image [92]. Therefore, the more uniform the distribution of the histogram of the encrypted image, the stronger the algorithm will be against statistical attacks. For experimental purposes, the “Lena.png” image was encrypted with an 8-bit RGB format with a size 512×512×3, therefore, it has a total of 786,432 pixels to be encrypted. Figure 12 shows the original image of Lena RGB 512×512×3 and the resulting cryptogram when implementing the encryption method with the HPCG generator by considering the states $x_{n+1}$ and $y_{n+1}$ of the Tinkerbell map [73] to generate the pseudo-random series. It is observed that the cryptogram is a totally unintelligible image that shows no traces of the original image, so it can be seen that the histograms of the RGB components have a uniform distribution, thus confirming that the cryptosystem is robust against statistical attacks [92,93].

#### 4.1.3. Entropy

In the works reported by Shannon [102,103], the mathematical foundations of the theory of information applied to communication and data storage were proposed. The entropy of the information is a criterion that measures the randomness of the data [13]. It can also be used to evaluate the security of the encryption [104]. Equation (5) is used to calculate the entropy H(s) [89,90,91], of a source (s),

(5) $$H\left(s\right)=\sum_{i=0}^{2^{N}-1}P\left(s_{i}\right)\cdot Log_{2}\left(\frac{1}{P(s_{i})}\right)\;\mathrm{bit},$$

where $P(s_{i})$ represents the probability of the $s_{i}$ symbol. For a purely random source that is emitting $2^{N}$ symbols with the same probability after evaluating (5), the entropy H(s)=N, in this case, for images with completely random pixels with 8-bit format, its ideal entropy is H(s)=8 bit. When digital images are encrypted, ideally their entropy must be 8. When a cryptographic system emits symbols (cryptograms) with entropy less than 8, this encryptor has a certain degree of predictability, so that its security is put at risk [13,89,90]. For purposes of comparison the entropy results to other related works that also report results using the Lena RGB image 512×512×3 with 8-bit RGB format. Table 2 shows that the proposed method using the Tinkerbell map [73] presents better entropy results versus most related works reported by [4,11,25,32,36,59,77,81], except work [41], which reports the better entropy. Thus, confirming that the high arithmetic precision helps to improve the entropy of the encrypted information.

On the other hand, using the FIAD cluster (see Figure 10 and Figure 11), Table 3 shows the entropy results obtained from the Lena RGB cryptogram of 512×512×3 using *n* process (n=1 to n=128) and the Tinkerbell map with high numerical precision np=99 [73]. It can be appreciated that in all the results there is an entropy of about 7.999xxxx, hence, it can be said that the cryptosystem’s security, i.e., entropy is not affected using parallel computing on embedded systems. In addition and with the purpose of testing the proposed method in a different hardware and with multiprocessing capability, the proposed method also was implemented in a computer with CPU AMD A6 4400M APU 2.7 GHz (Accelerated Processing Unit), which is a mobile dual-core processor based on the Trinity architecture, and it works with Windows 10 operating system and Python version 3.5.2.

#### 4.1.4. NPCR and UACI Differential Attacks

To perform an analysis against differential attacks and understand the differences between encrypted images [13], two measures in common are used, NPCR and UACI. These measures are used to test the influence of change of a pixel in the whole encrypted pattern.

Let us consider the cryptograms $C_{1}$ and $C_{2}$ obtained with a tiny difference of $1^{-90}$ in the encryption key using 1 processor obtained from a Lena RGB image with 8 bit RGB format adjusted to a size of M×N, where *M* = *N* = 512 pixels. According to [88], the critical values for the NPCR test in the image encryption with the levels of significance $N_{\alpha }$ are: $N_{0.05}=99.5994\%$, $N_{0.01}=99.5952\%$, and $N_{0.001}=99.5906\%$. Table 4 depicts the results of enforcement the NPCR test to the obtained cryptograms through the proposed method by implementing the Tinkerbell map [73]. If the values are less than $N_{\alpha }$, then, it is considered that $C_{1}$ and $C_{2}$ fail the test according to [88]. In Table 4, it can be observed that the results achieved by Tinkerbell map pass all the NPCR critical values test according to [88]. Also, it can be observed that the results obtained from NPCR have similar levels to those reported in related works [4,11,25,32,34,36,59,77].

Table 5 presents the NPCR results obtained with the Lena image 512×512×3 using FIAD cluster (see Figure 10 and Figure 11) with multiprocessors and the Tinkerbell map [73] for the encryption of the information. It can be observed that in most cases the test is passed according to the critical values established by [88], even though, some results failed by a hundredth or a thousandth before reaching the minimum critical value. Therefore, it can be said that the cryptosystem’s security is not affected by the use of parallel computing.

Table 6 shows the UACI results achieved with the Lena image 512×512×3 using FIAD cluster (illustrated in Figure 10 and Figure 11) and the Tinkerbell map [73] for the encryption information. It can be observed that in all cases, the UACI test is passed according to the critical values established by [88]. Therefore, it is verified that security is not affected by the use of parallel computing.

Regarding the security analysis performed on the cryptosystem against differential attacks NPCR and UACI, it can be concluded that it does not affect the use of more process in parallel for the encryption of information as shown in Table 4, Table 5 and Table 6, where the security levels of NPCR and UACI remain satisfactory regardless of the number of process used in encrypting the information.

### 4.2. Key Space

The key space is the total number of different keys that can be used in the encrypted or decrypted process [13]. For a cryptographic system to be effective and safe, the key space must be large enough to make unfeasible the brute force attack [92]. The key cryptosystem proposed consists of two parts: (i) the dynamic keys generator, and (ii) the control parameters of the same chaotic map. If the key space of an encryption algorithm is large enough, typically greater than 128 bits, it is already considered safe for most cryptographic applications in terms of the speed of current computers. According to the key space result reported in [73], the key space is $2^{2041}$ using the Tinkerbell map with a high precision of np=99 significant decimals. However, in this work it is proposed to use the FIAD cluster (illustrated in Figure 10 and Figure 11) the key space is increased virtually depending on the number *n* of process used. That is, the key space is $2^{n\times 2041}$, therefore, the brute force attack applied to the cryptosystem is unfeasible to break by current computers [13,93]. Table 7 lists the key space obtained by the proposed embedded cryptosystem using Tinkerbell chaotic map [73] and a comparison with other related works [4,25,32,33,35,36,37,38,39,40]. It can be observed, that there exists an exponential increase on the key space when using multiple precision in the numerical calculation determined by np=99 and np=999 significant decimals against the methods using double precision [4,25,32,33,35,36,37,38,39,40]. Hence, the Kerckhoffs’s principles are met, which says, the security of the system must rest on the security of the key, being supposed to know the rest of the parameters of the cryptosystem [37,38,105]. It can be appreciated that the key space results achieved in this paper using multiple precision are greater than those reported in related works [4,11,13,25,32,33,34,35,36,37,38,39,59,77]. Hence, the proposed embedded cryptosystem has virtually unlimited key space, such as [4,35,37,38,73].

### 4.3. Performance Results Using Parallel Computing

To execute the performance analysis using parallel computing and to ensure that it has an equitable distribution among the processors involved in the execution of the program in parallel, it is proposed to use the color image “lena.png” 320×320×3 with 8-bit format. Table 8 shows the results obtained on a FIAD cluster with six Raspberry Pi 3, Cortex-A-53 64-bit Quad Core 1.2 GHz processor, RAM memory 1 GB, and Raspbian Wheezy operating system, therefore, a total of 24 cores are integrated into the FIAD cluster. It shows the execution times of the sequential part α of the algorithm, as well as the execution times of the parts with parallel processing $\beta_{p}$ and communication time when the encryption algorithm is executed. In addition, it can be observed in Table 8 that the total encryption time using n=1 processor is 87.6573 s, and as *n* increases the number of process, the total time decreases until reaching a minimum time of 5.6330 s using n=16 process. Subsequently, the total time begins to increase, which means that the optimal number of process is n=16. This analysis allows discovery of the optimal number of processes and cores in parallel. In addition and with the purpose of testing the proposed method in a different hardware and with multiprocessing capability, the proposed method also was implemented in a personal computer with CPU AMD A6 4400M APU 2.7 GHz (Accelerated Processing Unit), which is a mobile dual-core processor based on the Trinity architecture, it works with Windows 10 operating system and Python version 3.5.2. With respect to the results obtained with the dual-core 2.7 GHz CPU, it is observed that the shortest encryption time is presented with n=2, this is due to the dual core of the hardware, that is, with this it is verified that they are necessary more cores to increase efficiency and reduce the total encryption time. In Table 8, it can be observed that as the number of processes increases, the speed of the PRNGs is increased directly proportional, this is because they work simultaneously.

**Figure 13:**
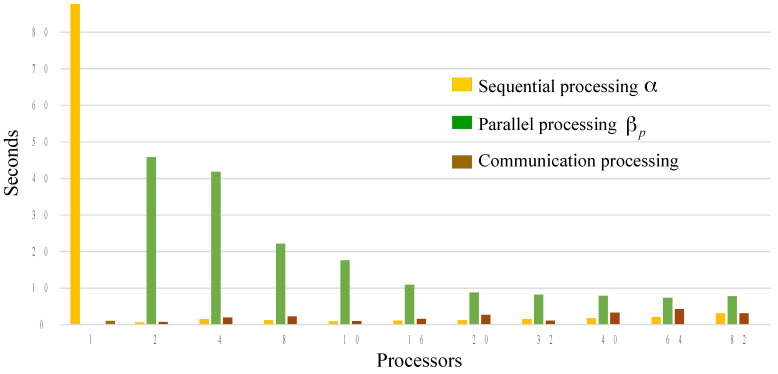
Performance graph of the proposed cryptographic method with parallel processing implemented in a FIAD cluster.

On the other hand, Figure 13 was obtained with the times of the FIAD cluster, it can be observed that the results obtained by encrypting the image of “lena.png ” in the FIAD cluster, the total encryption time using n=1 core is 87.6573 s and as the number of process increases, the total time decreases until it reaches a minimum of 5.6330 s with n=16 process. Hence, when using excess process in parallel without having the necessary hardware (cores), the performance is affected (these experiments were performed with a total of 24 cores). This verifies that the profiling tool helps to find the optimal number of process to be used in the encryption process in such a way that it is not only about increasing the process or using few processes for encryption—the interesting thing about the tool of profiling and processing of parallel computing is that it allows optimization of hardware resources.

**Figure 14:**
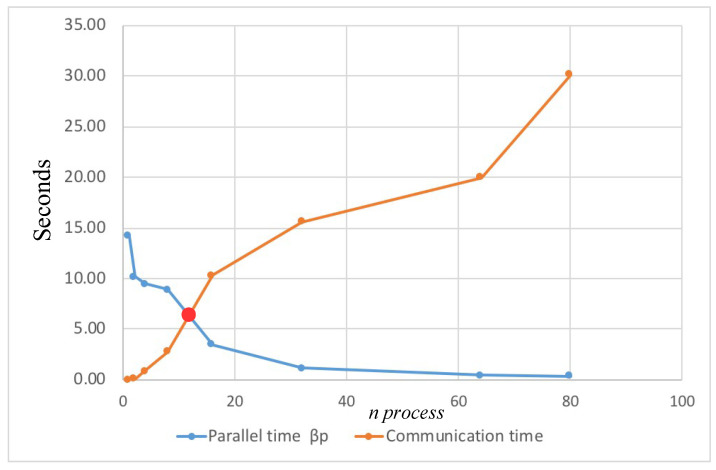
Performance graph of the proposed cryptographic method with parallel processing implemented in a Personal Computer Dual-core CPU 2.7 GHz.

Also, it is important to mention, in parallel computing, when the communication time is greater than the total processing time, it is said that the efficiency of the system begins to be lost, therefore there is no performance, in this case, it is recommended to increase the quantity of processors or cores, this can be observed in more detail in Figure 14.

**Figure 15:**
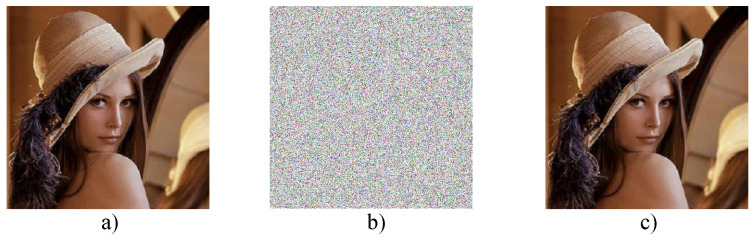
Result of encryption with parallel processing: (**a**) Input image, (**b**) Cryptogram, and (**c**) Recovered image.

Figure 15 shows the result of the encryption process of the input image “Lena.png”, where it can be observed that the recovered image Figure 15c is identical to the original image Figure 15a and that the cryptogram Figure 15b is totally unintelligible.

**Figure 16:**
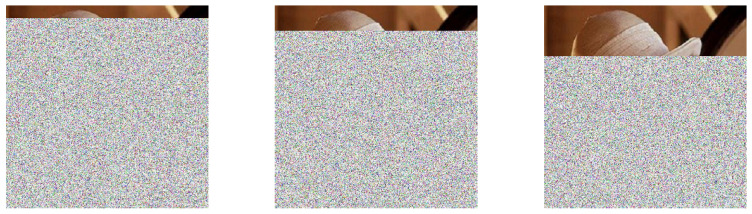
Examples of recovering the image in the process of decryption with an error in the parameter corresponding to the number of process.

In the same way, Figure 16 shows the result of the decryption process with an error in the parameter corresponding to the number *n* process involved in the encryption.

Finally, it is important to mention that when implementing the proposed method in a Raspberry Pi 3, the execution times of the program implemented with parallel processing are affected by the execution of other applications and services, so the theoretical estimations of the Performance factor and system efficiency may vary from real-time execution results. In addition, by encrypting the image separately (divided), *n* encryptions are performed where each encryption process has its corresponding encryption key (dynamic keys). Finally, it can be observed that the gain in execution time depends on the number of process involved in the execution of the program and the resource in software and hardware assigned to each processor, with n=16 process being the optimal value for the case of a FIAD cluster, which is within the total available cores (24 cores). In the case of the personal computer CPU 2.7 GHz, the optimal time was n=2, this because its hardware is a dual-core CPU.

## 5. Conclusions

In this paper, the implementation of a chaotic cryptosystem using profiling and parallel computation techniques in a cluster of embedded systems with multiprocessors was presented. With the experimental results, it was found that the efficiency of the embedded cryptosystem is improved and verified to comply with Amdahl’s law. To verify that the proposed method is scalable to other hardware with multiprocessing capability, the proposed method also was implemented in a personal computer with 2.7 GHz CPU, which is a mobile dual-core processor based on the Trinity architecture. In both hardware implementations, the results of security and performance analysis were satisfactory. The proposed method helps to find the optimal number of parallel processes to be used in the cryptosystem. It is verified that when using excess of processes in parallel without having the necessary hardware (cores), the performance is affected, as shown in Figure 13 and Figure 14. A great advantage of using parallel computing on embedded systems is that it is possible to reduce the total execution time by identifying the section of the algorithm that can be run simultaneously or parallel. In addition, it was possible to verify that the algorithms to generate the chaotic series can be adapted to obtain a high degree of parallelization. Using several processors and multiple precision in the cryptosystem add a greater degree of difficulty for cryptanalysts. Regarding the security analysis performed to the algorithm against the different types of attacks, such as statistical, entropy, differential (NPCR, UACI), key space, it can be concluded that the use of more processors in parallel for the encryption of the information does not affect, and as shown in Table 3, Table 4, Table 5, Table 6 and Table 7, the security levels remain satisfactory regardless of the *n* processes used. Regarding key space, when implementing the Tinkerbell map with high precision of np=99 significant decimals and when using *n* processes in parallel for the encryption of the information, the key space is increased virtually up to $2^{n\times 2041}$, where *n* is the number of parallel processes (see Table 7). Therefore, the Kerckhoffs’s principles are met. Finally, the proposed cryptographic method can be implemented in practical applications and with different types of hardware with multiprocessing capabilities.

## Figures and Tables

**Table 1:** NIST tests results for the chaotic sequences generated by each component of Tinkerbell map [73].

Test Name	*p*-Value			*p*-Value		
	*x*	Proportion	Results	*y*	Proportion	Results
Frequency	0.108791	0.980	Success	0.008513	0.960	Failed
Block Frequency	0.224821	1.000	Success	0.017694	1.000	Success
Cumulative Sums-Forward	0.304126	0.980	Success	0.009790	0.970	Failed
Cumulative Sums-Reverse	0.383827	0.980	Success	0.008536	0.970	Failed
Runs	0.009025	0.970	Failed	0.009200	0.970	Failed
Longest Run	0.554420	1.000	Success	0.224821	1.000	Success
Rank	0.129620	1.000	Success	0.851383	1.000	Success
FFT	0.595549	0.990	Success	0.924076	0.990	Success
Non-Overlapping Templates	0.455937	0.996	Success	0.637119	0.982	Success
Overlapping Templates	0.012971	0.990	Success	0.699313	0.990	Success
Universal	0.008285	0.970	Failed	0.012964	0.990	Success
Approximate Entropy	0.007955	0.960	Success	0.008597	0.970	Failed
Random Excursions	0.090936	0.996	Success	0.016196	0.985	Success
Random Excursions Variant	0.437274	0.998	Success	0.012650	0.986	Success
Linear Complexity	0.678686	0.990	Success	0.514124	0.990	Success
Serial (2m∇Ψ)	0.009240	0.975	Failed	0.955835	0.990	Success
Average	0.250716	0.985	-	0.306925	0.983	-

**Table 2:** Comparison of entropy results for Lena image of size 512×512×3 with 8-bit RGB format.

Cryptogram Size 512×512×3		Components RGB of 8 bit Entropy (bit/symbol)		
**Chaotic Map**	**Average Entropy**	**R**	**G**	**B**
Proposed Tinkerbell [73]	7.99925	7.99925	7.99924	7.99926
**Related work**				
Logistic 1D [25]	7.997200	7.99740	7.99750	7.99690
PELM [36]	7.994500	N/A	N/A	N/A
Tent [32]	7.998000	N/A	N/A	N/A
Novel 3D [4]	7.998200	7.99830	7.99820	7.99820
Chen [11]	7.997200	7.99720	7.99730	7.99710
Lorenz & Chen [81]	7.999300	N/A	N/A	N/A
5D Hyperchaotic [77]	7.997300	N/A	N/A	N/A
Logistic 1D [41]	7.999999	N/A	N/A	N/A
Logistic 1D [59]	7.999100	N/A	N/A	N/A

**Table 3:** Entropy for the cryptogram of Lena RGB image 512×512×3 using *n* processes and Tinkerbell map [73].

Cryptogram Size 512×512×3		Components RGB of 8 bit Entropy (bit/symbol)		
*n Process*	Average Entropy (%)	R	G	B
**FIAD cluster, CPU 1.2 GHz**				
1	7.9992743	7.9992716	7.9992623	7.9992888
2	7.9992962	7.9992525	7.9993085	7.9993277
4	7.9992476	7.9992258	7.9992211	7.9992959
8	7.9992692	7.9991658	7.9993238	7.9993182
16	7.9992042	7.9991641	7.9992064	7.9992421
32	7.9992992	7.9991472	7.9993798	7.9993705
64	7.9992831	7.9992403	7.9993189	7.9992901
128	7.9993141	7.9992695	7.9992722	7.9994006
**CPU 2.7 GHz**				
1	7.9992742	7.9992716	7.9992623	7.9992888
2	7.9992962	7.9992525	7.9993085	7.9993276
4	7.9992476	7.9992258	7.9992210	7.9992959
8	7.9992692	7.9991657	7.9993237	7.9993181
16	7.9992041	7.9991640	7.9992063	7.9992421
32	7.9992991	7.9991472	7.9993798	7.9993704
64	7.9992831	7.9992403	7.9993188	7.9992901
128	7.9993140	7.9992695	7.9992721	7.9994006

**Table 4:** NPCR test for the cryptogram of Lena image 512×512×3 pixels using 1 processor and Tinkerbell map [73], and comparison versus other related work.

Cryptogram Size		Critical NPCR Values [88]		
512×512×3		$N_{0.05}$ = 99.5893%,	$N_{0.01}$ = 99.5810%,	$N_{0.001}$ = 99.5717%,
**Chaotic Map**	**Average NPCR** (%)	**NPCR Test Result**		
Proposed Tinkerbell [73]	99.6029	Passed	Passed	Passed
**Related work**				
Logistic 1D [25]	99.6100	Passed	Passed	Passed
PELM [36]	99.5774	Failed	Failed	Passed
Multi-modal [34]	99.0000	Failed	Failed	Failed
Tent map [32]	99.6300	Passed	Passed	Passed
Novel 3D [4]	99.6100	Passed	Passed	Passed
Chen [11]	99.8031	Passed	Passed	Passed
5D Hyperchaotic [77]	99.6122	Passed	Passed	Passed
Logistic 1D [59]	99.6100	Passed	Passed	Passed

**Table 5:** NPCR test for cryptogram of Lena image 512×512×3 pixels using *n* processes and Tinkerbell map [73].

Cryptogram Size		Critical NPCR Values [88]		
512×512×3		$N_{0.05}$ = 99.5893%,	$N_{0.01}$ = 99.5810%,	$N_{0.001}$ = 99.5717%,
*n Process*	Average NPCR(%)	NPCR Test Result		
**FIAD cluster (1.2 GHz)**				
1	99.6029	Passed	Passed	Passed
2	99.5925	Passed	Passed	Passed
4	99.5900	Passed	Passed	Passed
8	99.5908	Passed	Passed	Passed
16	99.5884	Failed	Passed	Passed
32	99.5930	Passed	Passed	Passed
64	99.5908	Passed	Passed	Passed
128	99.5749	Failed	Failed	Passed
**CPU (2.7) GHz**				
1	99.6028	Passed	Passed	Passed
2	99.5924	Passed	Passed	Passed
4	99.5900	Passed	Passed	Passed
8	99.5908	Passed	Passed	Passed
16	99.5883	Failed	Passed	Passed
32	99.5929	Passed	Passed	Passed
64	99.5908	Passed	Passed	Passed
128	99.5749	Failed	Failed	Passed

**Table 6:** UACI test for cryptogram of Lena image 512×512×3 pixels using *n* processes with Tinkerbell map [73] and comparison versus other related work.

Cryptogram Size		Critical UACI Values [88]		
512×512×3		$U_{0.05}^{-}$ = 33.3730%, $U_{0.05}^{+}$ = 33.5541%,	$U_{0.01}^{-}$ = 33.3445%, $U_{0.01}^{+}$ = 33.5826%,	$U_{0.001}^{-}$ = 33.3115%, $U_{0.001}^{+}$ = 33.6156%,
*n Process*	Average UACI (%)	UACI Test Result		
**FIAD cluster, CPU 1.2 GHz**				
1	33.4318	Passed	Passed	Passed
2	33.4113	Passed	Passed	Passed
4	33.4262	Passed	Passed	Passed
8	33.4206	Passed	Passed	Passed
16	33.4452	Passed	Passed	Passed
32	33.4431	Passed	Passed	Passed
64	33.4814	Passed	Passed	Passed
128	33.4140	Passed	Passed	Passed
**CPU (2.7) GHz**				
1	33.4317	Passed	Passed	Passed
2	33.4112	Passed	Passed	Passed
4	33.4262	Passed	Passed	Passed
8	33.4205	Passed	Passed	Passed
16	33.4452	Passed	Passed	Passed
32	33.4431	Passed	Passed	Passed
64	33.4814	Passed	Passed	Passed
128	33.4139	Passed	Passed	Passed
**Related work**				
Logistic 1D [25]	33.3600%	Failed	Passed	Passed
PELM [36]	33.3014%	Failed	Failed	Failed
Multi-modal [34]	34.8353%	Failed	Failed	Failed
Tent map [32]	33.2800%	Failed	Failed	Failed
Novel 3D [4]	33.4500%	Passed	Passed	Passed
Chen [11]	33.6236%	Failed	Failed	Failed
5D Hyperchaotic [77]	33.4573%	Passed	Passed	Passed
Logistic 1D [59]	33.4500%	Passed	Passed	Passed

**Table 7:** Comparison of key space to other related works.

Key Space				
**Chaotic** **Map**	**Simple Precision** np=8	**Double Precision** np=16	**Multiple Precision** **(Proposed PRNG)**	
			np=99	np=999
Proposed Tinkerbell (n process) [73]	$2^{n\times 192}$	$2^{n\times 384}$	$2^{n\times 2041}$	$2^{n\times 19,979}$
Tinkerbell (1−process) [73]	$2^{192}$	$2^{384}$	$2^{2041}$	$2^{19,979}$
**Related work**				
Rössler hyperchaotic [13]	N/A	$2^{744}$	N/A	N/A
Discrete [35]	N/A	$2^{428}$	N/A	N/A
Three chaotic maps [37]	N/A	$2^{400}$	N/A	N/A
5D Hyperchaotic [77]	N/A	$10^{90}≃2^{299}$	N/A	N/A
Chen [11]	N/A	$2^{256}$	N/A	N/A
Tent map [32]	N/A	$2^{255}$	N/A	N/A
Two chaotic maps [38]	N/A	$2^{213}$	N/A	N/A
Logistic 1D [59]	N/A	$2^{199}$	N/A	N/A
Novel 3D [4]	N/A	$2^{198}$	N/A	N/A
Tinkerbell [39]	N/A	$2^{183}$	N/A	N/A
Multi-modal [34]	N/A	$2^{159}$	N/A	N/A
PLM [33]	N/A	$2^{136}$	N/A	N/A
Logistic 1D [36]	N/A	$2^{128}$	N/A	N/A
Logistic 1D [25]	N/A	$2^{128}$	N/A	N/A

**Table 8:** Comparison of performance with other related work.

Quantity (*n Process*)	Cryptosystem Times and PRNG Speed				
	Total Time (s)	α (s)	$\beta_{p}\left(s\right)$	Communication Time (s)	PRNG Speed (Mbit/s)
**FIAD cluster, CPU 1.2 GHz**					
1	87.6573	87.6563	N/A	N/A	0.011799
2	46.7736	0.5612	45.8394	0.3730	0.045848
4	42.6746	1.4609	41.7796	1.0299	0.100242
8	24.7102	1.1856	22.0905	1.4341	0.381060
10	15.3867	0.9041	15.3251	3.0074	0.682574
16	5.6330	1.1036	5.6104	6.1188	2.998515
20	5.7472	1.2210	4.5173	6.0029	4.673369
32	6.2665	1.5226	5.4581	4.6424	6.169972
40	7.7156	1.7689	6.7280	3.0649	6.338776
64	7.6150	2.0601	3.2211	7.4354	20.131226
80	7.9121	3.0578	6.6620	3.3478	12.362623
**Dual core CPU 2.7 GHz**					
1	14.2769	14.2769	N/A	N/A	0.114700
2	10.3276	0.1136	10.1526	0.0614	0.317600
4	10.5160	0.2208	9.4552	0.8400	0.580280
8	12.1092	0.3753	8.9377	2.7962	1.259200
16	14.0868	0.3293	3.4450	10.3125	7.654400
32	18.0704	1.3010	1.1705	15.5989	44.56640
64	21.5714	1.1648	0.4477	19.9589	199.6928
80	31.4447	0.9118	0.3797	30.1532	348.5520
**Related work**					
1 CPU, 2.7 GHz [73]	N/A	N/A	N/A	N/A	9.1491
1 CPU, 2.5 GHz [32]	N/A	N/A	N/A	N/A	0.1450
1 CPU 2.0 GHz [36]	N/A	N/A	N/A	N/A	1.7000
1 CPU, 1.9 GHz [35]	N/A	N/A	N/A	N/A	1.2600
1 CPU, 2.8 GHz [39]	N/A	N/A	N/A	N/A	0.4901
1 CPU, 2.1 GHz [39]	N/A	N/A	N/A	N/A	0.4844
1 CPU, 2.4 GHz [11]	N/A	N/A	N/A	N/A	47.7653
1 CPU, 3.0 GHz [15]	N/A	N/A	N/A	N/A	44.9389
Octa core CPU, 2.5 GHz [41]		N/A	N/A	N/A	118.90
